# Bioengineered intestinal tubules as a tool to test intestinal biological efficacy of lettuce species

**DOI:** 10.1038/s41538-022-00175-x

**Published:** 2022-12-13

**Authors:** Paulus G. M. Jochems, Bo Heming, Dmitry Lapin, Naomi E. L. Moonen, Guido Van den Ackerveken, Rosalinde Masereeuw

**Affiliations:** 1 Division of Pharmacology, Utrecht Institute for Pharmaceutical Sciences, Utrecht University, Utrecht, The Netherlands; 2 Translational Plant Biology, Department of Biology, Utrecht University, Utrecht, The Netherlands; 3 Jheronimus Academy of Data Science, s’ Hertogenbosch, The Netherlands

**Keywords:** Physiology, Nutrition disorders

## Abstract

Lettuce (*Lactuca sativa*) is one of the most consumed and cultivated vegetables globally. Its breeding is focused on the improvement of yield and disease resistance. However, potential detrimental or beneficial health effects for the consumer are often not targeted in the breeding programs. Here, a bioengineered intestinal tubule was used to assess the intestinal efficacy of extracts from five plant accessions belonging to four *Lactuca* species. These four species include the domesticated *L. sativa*, closely related wild species *L. serriola*, and phylogenetically more distant wild relatives *L. saligna* and *L. virosa*. We assessed the epithelial barrier integrity, cell viability, cell attachment, brush border enzyme activity, and immune markers. Extracts from *L. sativa* cv. Salinas decreased cell attachment and brush border enzyme activity. However, extracts from the non-edible wild species *L. saligna* and *L. virosa* reduced the epithelial barrier functions, cell attachment, cell viability, and brush border enzyme activity. Since wild species represent a valuable germplasm pool, the bioengineered intestinal tubules could open ways to evaluate the safety and nutritional properties of the lettuce breeding material originating from crosses with wild *Lactuca* species.

## Introduction

With its primary role in the digestion and absorption of nutrients, the intestine is one of the first organ systems where food can exert biological effects. Food safety and efficacy studies are frequently performed in animals that are costly, time-consuming, subject to ethical debate, and low throughput^1^. Microphysiological models based on organ-on-a-chip technologies could help overcome these issues. These models recapitulate the physiological microenvironment of an organ more closely compared to current gold standard in vitro models, which often lack *e.g*. differentiation into specialized epithelial cell types^2^. We recently developed a bioengineered intestinal tubule with enterocytes, goblet, Paneth, enteroendocrine, and stem cells that resembles the intestine heterogeneous cell population more closely than the gold standard Transwell™ model^3^. The bioengineered tubule mimics the three-dimensional tube-like configuration of the intestine, including villi structures, and it is compatible with transepithelial transport assessments^3,4^. In contrast to most organ-on-a-chip models that are being applied in biomedicine and drug research, this bioengineered intestinal tubule was validated to assess the safety and efficacy of nutrition. During these assessments, the integrity of the intestinal epithelial barrier using membrane-associated complexes of zonula occludens-1 (ZO-1) as a marker, overall cell viability, activity of the brush border enzyme alkaline phosphatase, which is essential for the final steps of digestion, and the release of nitric oxide (NO) and cytokines as markers of the immune status were tested upon exposure to food proteins^5–8^.

Plant breeding is crucial for improving crop yield and quality^9^. Lettuce (*Lactuca sativa*) is one of the leading global leafy vegetables in the production and value^10^. Its breeding is focused primarily on disease resistance, marketable head size, leaf shape and color, resistance to bolting, and bitterness^11^. Wild relatives of the domesticated lettuce, *L. serriola*, *L. saligna*, and *L. virosa*, are assigned to three gene pools (GPs), GP1, GP2, and GP3, respectively, based on their genetic relatedness to the domesticated lettuce *L. sativa*^12^. Wild relatives of domesticated lettuce are valuable sources of disease resistance traits, abiotic stress tolerance, and plant architecture traits^13^. Lettuce leaves consist of ~95% water, and dry matter contains bioactive compounds such as folate, vitamin C, vitamin E, short-chain fatty acids, carotenoids, phenolic acid, and flavonoids^14^. Metabolic profiling of *Lactuca* accessions from different GPs revealed species-correlated patterns in the metabolite composition. Specifically, wild relatives of cultivated lettuce, *L. serriola*, *L. saligna*, and *L. virosa*, have a high content of bitterness-related compounds such as jacquinelin and lactucopicrin^12^. A reduction in the content of anti-nutritional and bitter-tasting molecules has also happened during the domestication of other crops like tomato and potato^15,16^. Although metabolome-assisted selection combined with machine learning could become a viable option in the vegetable and fruit breeding^17^, testing for the potential toxicity of breeding material is more challenging.

Here, we first used bioengineered intestinal tubules to evaluate extracts from commercially produced lettuce. We assessed parameters of intestinal biological efficacy such as epithelial barrier integrity, cell viability, cell attachment, alkaline phosphatase activity, NO accumulation, and the secretion of interleukin (IL)-6 and IL-8. Next, we compared *L. sativa* cultivars Salinas and Olof, *L. serriola* US96UC23 (GP1)*, L. saligna* CGN05271 (GP2), and *L. virosa* CGN04683 (GP3) in the tubules using the same test parameters. In contrast to extracts from *L. sativa* and *L. serriola* lines grown under controlled laboratory conditions, accessions of wild species *L. saligna* and *L. virosa* had detrimental effects on epithelial barrier integrity and cell viability. These observations indicate that the bioengineered intestinal tubule could be used as an organ-on-chip system to evaluate lettuce germplasm and crosses for toxicity.

## Results

### Extracts from commercially available lettuce material do not disrupt the epithelial barrier

**Fig. 1:**
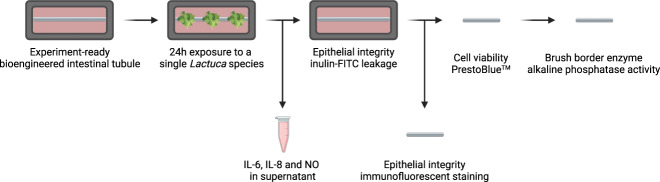
Schematic representation of the experimental procedure. Bioengineered intestinal tubules were exposed to crude extracts from *Lactuca* for 24 h in the cell cultivation medium. The supernatant was collected to assess interleukin (IL)-6, IL-8, and nitric oxide (NO) content. The epithelial integrity was determined after removing the supernatant using the inulin-FITC leakage test. After removing the hollow fiber membrane from the chamber, the tubule was assessed for epithelial integrity via immunofluorescent staining or alkaline phosphatase activity and cell viability. Created with BioRender, license 6221c9168684e9004c47503b.

To test the bioengineered intestinal tubule as a system to assess intestinal biological efficacy of *Lactuca* lines, we first used four commercially available and commonly consumed lettuce types, referred to as the validation lettuce material. This validation set included leaf material of butterhead, lollo rosso, red iceberg lettuce, and the stem of stalk lettuce that were ground to a fine powder in liquid nitrogen. The powder was added to the culture medium. After the 24 h exposure, we measured the intestinal epithelial integrity by assessing inulin-FITC leakage and ZO-1 expression, cell viability, cell number, alkaline phosphatase activity, IL-6 secretion, IL-8 secretion, and NO content **(**Fig. 1**)**.

**Fig. 2:**
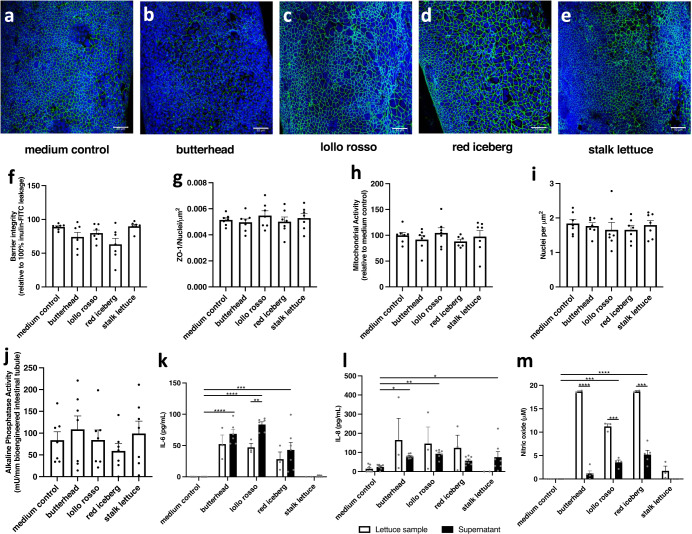
Extracts from commercially available lettuce material do not disrupt the epithelial barrier or reduce cell viability in bioengineered intestinal tubules after 24 h exposure. **a**–**e** Representative immunofluorescent images of nuclei (blue) and zonula occludens-1 (ZO-1, green) for medium control, butterhead, lollo rosso, red iceberg, and stalk lettuce exposed bioengineered intestinal tubules, respectively. **f**, **g** Assessment of the intestinal epithelial barrier via inulin-FITC permeability **f** and ZO-1 quantification **g**. Inulin-FITC permeability data are expressed as a percentage (%) of the negative control, and ZO-1 quantification data were corrected for surface area and the number of nuclei. **h** The mitochondrial activity is a measure of cell viability. Data are relative to the cell cultivation medium without plant extracts. **i** The number of nuclei corrected for the surface area as a measure of cell attachment. **j** Brush border enzyme activity of alkaline phosphatase, a common Caco-2 enterocyte differentiation marker. **k**–**m** The secretion of interleukin (IL)-6, IL-8 and nitric oxide (NO), respectively. White bars (*n* = 3) represent the concentration in lettuce extracts without exposure to tubules, whereas black bars visualize the concentration after 24 h exposure. Data are shown as mean ± SEM of *n* = 7 independent experiments. Data were corrected for outliers (6 outliers in 354 data points) and tested for significance using a t-test and one-way ANOVA. *p < 0.05, **p < 0.005, ***p < 0.001 and ****p < 0.0001. Scale bar is 50 µM.

In bioengineered intestinal tubules exposed to the medium without lettuce extracts, we observed a viable and functional epithelial barrier. Levels of alkaline phosphatase and the immune markers, IL-6, IL-8 and NO, remained at baseline levels (Fig. 2) as expected^3^. Previously shown characteristics of the bioengineered intestinal tubule were further confirmed by positive staining and visualization of the epithelial cell differentiation towards goblet cells (Supplementary Fig. 1a) and three-dimensional morphology, including villi structures (Supplementary Fig. 1b). In comparison to the medium-only control, exposure to the validation lettuce material did not affect the intestinal epithelial integrity (Fig. 2a–g), cell viability (Fig. 2h), cell attachment (Fig. 2i), and alkaline phosphatase activity (Fig. 2j). Hence, intestinal epithelial cells remained functional and viable upon exposure to the validation lettuce material. We also quantified the immune markers IL-6, IL-8, and NO in the extract-exposed bioengineered intestinal tubule (Fig. 2k–m). Background IL-6 and IL-8 signals were detected in the plant extracts before exposure to the tubule, and the IL-6 secretion has slightly increased after exposure to lollo rosso extracts (Fig. 2k). The NO levels in the supernatant of extract-exposed tubules were lower than in the starting extracts of butterhead, lollo rosso, and red iceberg leaves themselves (Fig. 2m). Thus, we cannot separate the response of the intestine cells from the background measurements in the lettuce extracts for the tested immune markers. We concluded that while secreted IL-6, IL-8, and NO are likely not suitable to study the biological efficacy of lettuce extracts, cell viability and attachment, as well as the epithelial barrier integrity in the bioengineered intestinal tubules could be used as readouts for checking plant toxicity.

### Extracts from wild lettuce relatives L. saligna and L. virosa reduce epithelial integrity and cell viability in the bioengineered intestinal tubule

**Fig. 3:**
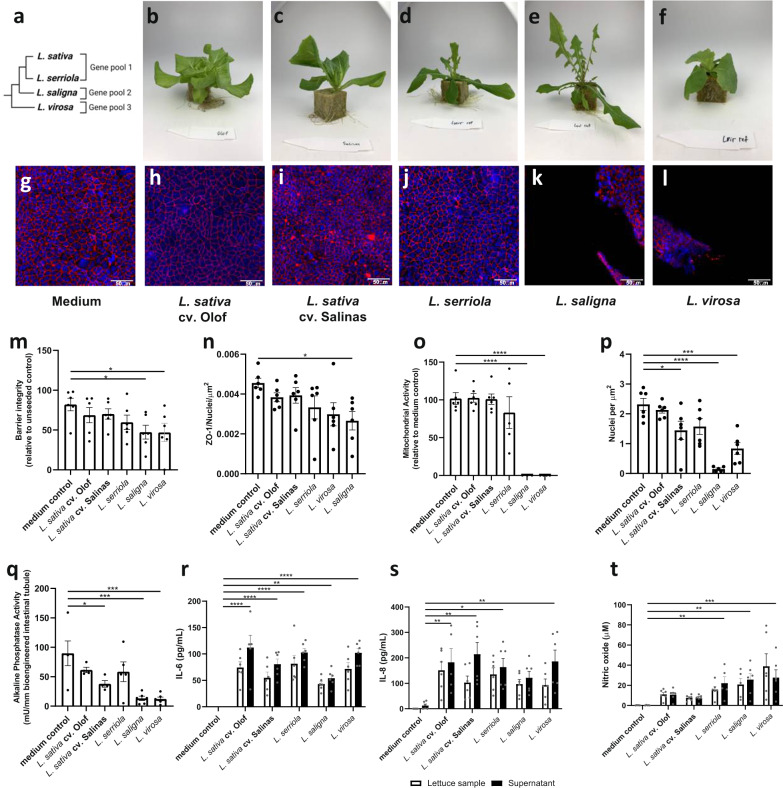
Extracts from wild lettuce relatives *L. saligna* and *L. virosa* reduce epithelial integrity and cell viability in the bioengineered intestinal tubule. **a** Schematic of the phylogenetic tree for *Lactuca* species. *L. sativa* and wild progenitor belong to gene pool 1, while *L. saligna* and *L. virosa* are from pools 2 and 3, respectively. Representative photographs of 2.5–3-week-old plants of **b** *L. sativa* cv. Olof, **c** *L. sativa* cv. Salinas, **d** *L. serriola* US96UC23, **e** *L. saligna* CGN05271, **f** *L. virosa* CGN04683 used in the study. (G-L) Representative immunofluorescent images of nuclei (blue) and zonula occludens-1 (ZO-1, red) on intestinal tubule after exposure to medium (control) or extracts of *L. sativa* cv. Salinas and *L. sativa* cv. Olof, *L. serriola*, *L. saligna*, and *L. virosa*, respectively. **m**, **n** The intestinal epithelial barrier integrity is assessed via inulin-FITC permeability **m** and ZO-1 quantification **n**. Inulin-FITC permeability data are shown relative to the negative control, and ZO-1 quantification data are corrected for surface area and the number of nuclei. **o** Mitochondrial activity as a measure of cell viability after exposure to *Lactuca* extracts. Data are shown relative to medium control. **p** The number of nuclei corrected for the surface area as a measure of cell attachment. **q** Brush border enzyme activity of alkaline phosphatase, a common Caco-2 enterocyte differentiation marker. **r**–**t** Amounts of the secreted immune markers interleukin (IL)-6, IL-8, and nitric oxide (NO), respectively. White bars represent basal levels in the *Lactuca* extracts, whereas black bars visualize the concentration in the supernatant after 24 h of cellular exposure. Data are presented as mean ± SEM of *n* = 6 independent experiments. Data were corrected for outliers (1 outlier of 402 data points) and tested for significance using a t-test and one-way ANOVA. *p < 0.05, **p < 0.005, ***p < 0.001 and ****p < 0.0001. Scale bar is 50 µM.

Next, we evaluated wild *Lactuca* species alongside *L. sativa* in the bioengineered intestinal tubules at the level of the intestinal epithelial integrity, cell viability, cell attachment, alkaline phosphatase activity, secretion of IL-6 and IL-8 secretion, and the NO content (Fig. 3). Three tested wild *Lactuca* species show different levels of relatedness to domesticated lettuce *L. sativa* (Fig. 3a–f). *L. serriola*, or prickly lettuce, is the closest tested relative that produces fertile progeny with *L. sativa* (gene pool GP1), and *L. saligna* (GP2) with *L. virosa* (GP3) are more distantly related.

Exposure of the tubules to shoot extracts of ~2.5-week-old *L. sativa* cv. Salinas, *L. sativa* cv. Olof and *L. serriola* US96UC23 did not significantly affect epithelial barrier integrity (Fig. 3g–j). This was evident from the inulin-FITC permeability measurements (Fig. 3m) and the abundance of plasma membrane protein ZO-1, which limits the movement of substances across the paracellular space (Fig. 3n). By contrast, extracts from *L. saligna* CGN05271 and *L. virosa* CGN04683 resulted in a significant decrease in epithelial barrier integrity compared to the medium control (Fig. 3k–m). ZO-1 density was reduced significantly only for extracts from the *L. saligna* accession (Fig. 3n). Consistent with epithelial barrier integrity assays, intestinal epithelial cells exposed to extracts from the tested *L. virosa* and *L. saligna* accessions completely lost mitochondrial activity, a marker of cell viability (Fig. 3o). We observed a decrease in the number of attached epithelial cells in the presence of extracts from *L. virosa*, *L. saligna*, and to a lower extent with extracts *L. sativa* cv. Salinas and *L. serriola* (Fig. 3l, p). A similar pattern was measured for alkaline phosphatase activity after exposures to *L. sativa* cv. Salinas, *L. saligna*, and *L. virosa* (Fig. 3q). As seen with the validation set (Fig. 2k–m), increased levels of secreted IL-6/IL-8 and NO were measured in the bioengineered intestinal tubules exposed to the *Lactuca* extracts (Fig. 3r–t, black bars). However, these values were similar to the plant extracts on their own (Fig. 3r–t, white bars). Thus, extracts from *L. saligna* CGN05271 and *L. virosa* CGN04683 affect cell viability and disrupt the integrity of the epithelial barrier stronger than tested lines from GP1, the domesticated lettuce and its close relative *L. serriola* US96UC23.

**Fig. 4:**
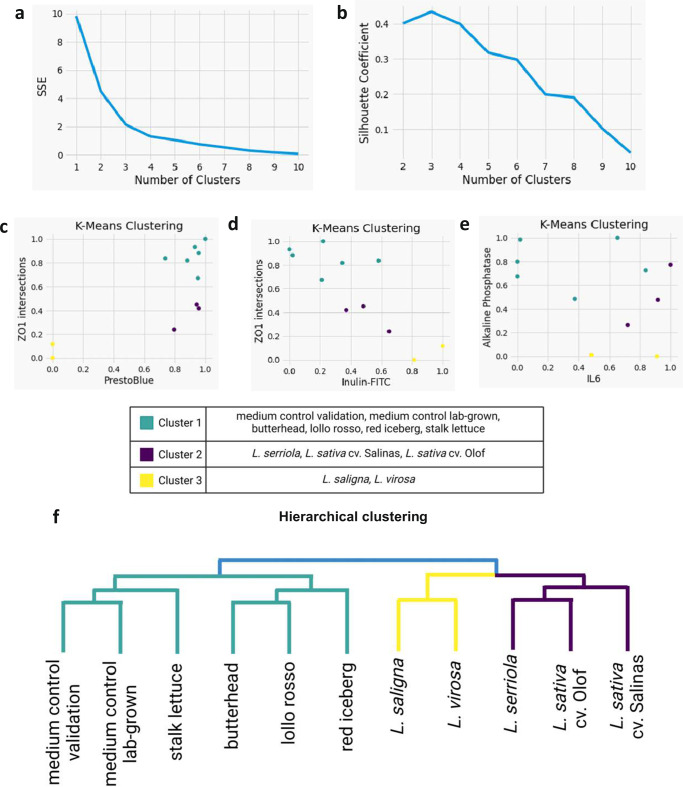
Comprehensive analyses reveal distinct intestinal biological behavior of *Lactuca* lines at different stages of domestication. **a** shows the sum of squares due to error (SSE) to find an optimal number of clusters. **b** shows the evaluation of the silhouette coefficient. **c**–**e** shows three different K-means visualizations of the clustering. **f** illustrates the results of a hierarchical clustering method.

Finally, we compared the results of the validation set, consisting of commercially available lettuce (Fig. 2), and the *Lactuca* species grown in the laboratory (Fig. 3). Mean biological efficacy parameters scaled from 0 to 1 were clustered using the k-means hierarchical clustering approach (Fig. 4). The optimal number of clusters was determined by the sum of squared errors (SSE) (Fig. 4a). This is the sum of the squared differences between each cluster’s mean and the observations for individual lines within this cluster. The SSE curve flattened around an optimal number of clusters of three-four (Fig. 4a). The silhouette coefficient supported this result of optimal separation of samples in three groups (Fig. 4b). Cluster 1 consisted of the two medium control samples and the four commercially available lettuce lines from the validation set. Lettuce material in this cluster showed a negligible effect on bioengineered intestinal tubules (Fig. 4c–f). Cluster 2 clustered the laboratory-grown lettuce lines originating from GP1, *L. sativa* cv. Salinas, *L. sativa* cv. Olof, and *L. serriola* (Fig. 4c–f). Cluster 3 with wild *L. saligna* and *L. virosa* from GP2 and GP3, respectively (Fig. 4c–f), was characterized by reduced cell viability (mitochondrial and alkaline phosphatase activities) and decreased epithelial barrier integrity (ZO-1 intersects, inulin-FITC permeability) (Fig. 4c–e). Thus, the separation of laboratory-grown *Lactuca* lines based on their intestinal biological efficacy aligned with the crop domestication level (Fig. 3a).

## Discussion

The organ-on-chip models maintain important physiological characteristics of respective organs necessary for their appropriate application to study biological phenomena. The bioengineered intestinal tubule accurately recapitulates the functional and viable intestinal epithelium, differentiating epithelial cells morphologically resembling the in vivo organ, and low levels of immunity markers such as secreted IL-6 and IL-8, NO levels^3^. We used this model to analyze the biological efficacy of extracts from cultivated lettuce and its wild relatives. Expectedly, intestinal epithelial barrier, cell viability, and brush border enzyme activity were preserved upon exposure of the tubules to lettuce from the supermarket (Fig. 2). Similarly, extracts from cultivated lettuce genotypes or the closely related wild relative *L. serriola* grown in the laboratory did not affect cell viability or integrity of the epithelial barrier of the model (Fig. 3m–o). In contrast, extracts from two distant wild relatives of the cultivated lettuce *L. saligna* and *L. virosa* were detrimental to these parameters in the model (Fig. 3k, l, o–q). Whether heterogeneity of cell populations in the tubules is essential for evaluation purposes is to be determined in future studies.

We detected nitric oxide (NO) and secreted IL-6 and IL-8 not only in the supernatants of lettuce-exposed tubules but also in the starting lettuce extracts (Figs. 2k–m, 3r–t). This is likely caused by the cross-reactivity of antibodies against IL-6 and IL-8 with plant proteins. Since NO is also present in plants^18^, we find the value of IL-6, IL-8, and NO as immune markers for testing the biological efficacy of lettuce extracts limited. However, IL-6 was increased upon lollo rosso exposure and NO was decreased upon butterhead, lollo rosso and red iceberg exposure. The latter is potentially due to the absorptive capacity of the intestinal cells. Still, IL-6 can induce intestinal inflammation and increase epithelial permeability^19,20^. Therefore, we compared IL-6 concentrations in our assays with other publications. In vitro modeling of intestinal inflammation indicates IL-6 concentration 50–80 pg/mL under physiological conditions and up to 700 pg/mL under inflammatory conditions^21,22^. Values measured in our experiments after adding lettuce extracts (<150 pg/mL) are close to physiological levels (Figs. 2 and 3). Whether lettuce has immunomodulating properties in the bioengineered intestinal tubule remains unclear due to the background IL-6/IL-8 measurements in the plant extracts. In future studies, additional evaluation of gene expression, correlating these to protein secretion data, in the intestinal epithelial cells can help to clarify the potential immunomodulatory properties of lettuce.

Phytochemical profiles of lettuce are thought to play an essential role in the induction of health effects^23^. In previous studies, metabolomic profiling of *Lactuca* accessions identified ~1900 chemicals, 264 of which were present in *L. serriola*, *L. saligna*, and *L. virosa* but absent in *L. sativa*^12^. Breeding towards favorable traits *e.g*., reduced bitterness, alters phytochemical profiles and it is associated with a reduction in quinate, chlorogenic acid, and sesquiterpene lactones (SLs)^23–25^. We found that the intestinal barrier integrity and cell viability of the bioengineered intestinal tubules were higher in extracts from cultivated lettuce lines than the wild *Lactuca* species *L. saligna* and *L. virosa* (Fig. 3k, l, o–q). This could be explained by the increased levels of fumarate and *myo*-inositol in cultivated lettuce compared to wild accessions^24,26^. The fumarate derivative, dimethyl-fumarate (DMF), has anti-inflammatory and antioxidant capacity and it can also improve the tight-junction protein expression^27^. *Myo-*inositol is a precursor of phosphoinositides and inositol phosphates such as phytic acid (PA). Animal studies showed a barrier protective effect of PA in a colorectal cancer model, possibly due to preserved stability of the E-cadherin/β-catenin complex essential for cell-cell adhesion^28^. On the other hand, high levels of SLs in *L. saligna* and *L. virosa* might be responsible for the observed toxicity. These phytochemicals have demonstrated biological effects in the gastrointestinal tract and possess antimicrobial properties^29^. Guaianolide SLs could also induce apoptosis via the ROS mitochondrial pathway in Caco-2 cells^30^. Nevertheless, further research is needed to link *Lactuca* metabolic profiles to the biological efficacy and health effects of lettuce and its wild relatives.

### Comprehensive analyses reveal distinct intestinal biological behavior of *Lactuca* lines at different stages of domestication

Comprehensive approaches to analyze the nutritional properties of food could help combine different measured parameters and inform the decision-making^31^. Clustering analysis of biological efficacy assessments in this study (Fig. 4) separated all analyzed materials into three groups: (i) commercially produced lettuce from the supermarket (‘validation set’), (ii) young (~2.5 weeks) domesticated lettuce and its close wild relative cultivated in the laboratory, and (iii) young (~2.5 weeks) laboratory grown distant lettuce relatives *L. saligna* and *L. virosa*. Although growth conditions like nitrogen fertilizer and light conditions as well as age impact phytochemical profiles, it is difficult to pinpoint what drives the separation of the validation set from the other samples^32,33^. At the same time, separation of *L. sativa* and *L. serriola* vs *L. saligna* and *L. virosa* accessions was clearly driven by adverse effects of the latter on cell viability and epithelial barrier integrity in the bioengineered intestinal tubules. Importantly, *Lactuca* lines were cultivated in the laboratory simultaneously, side by side, ensuring that the differences are genetically driven. Earlier studies reported toxicity of the directly consumed *L. virosa* and analgetic or sedative effects of *L. virosa*-derived compounds^34,35^. Although *L. saligna* and *L. virosa* are valuable resources of genetic variation in lettuce breeding^13^, lettuce cultivars are not tested routinely for potential beneficial or detrimental nutritional effects. Intestinal organ-on-chip models and holistic analysis of their viability and epithelial barrier integrity in the presence of plant extracts could open opportunities to develop lettuce varieties with improved nutritional properties.

## Methods

### Chemicals

Unless stated otherwise, all chemicals were purchased from Sigma-Aldrich (Zwijndrecht, The Netherlands).

### Caco-2 cell culture

The human colon adenocarcinoma-derived intestinal cell line, Caco-2 (ATCC, Wesel, Germany), was maintained in high glucose Dulbecco’s Modified Eagle Medium–high glucose (Gibco, Bleiswijk, The Netherlands) supplemented with fetal calf serum (10% v/v) and penicillin and streptomycin (1% v/v). Media was refreshed every 2–3 days, and cells were passaged and seeded on bioengineered intestinal tubules when reaching 80–90% confluency.

### Bioengineered intestinal tubule

The construction, extracellular matrix (ECM) coating, seeding, and cultivation of the bioengineered intestinal tubule were described before and performed according to^36^. In short, a hollow fiber membrane (HFM) was guided in a custom-made 3-dimensional printed polylactic acid chamber. 18-gauge blunt needles (OctoInkjet, Hoyland, United Kingdom) were placed inside the in- and outlet of the chamber to enable perfusion experiments. Leak-tightness of the chamber was guaranteed by biocompatible glue, GI-MASK Automix glue (Coltene, Lezennes, France). Finally, the bottom of the chamber was sealed using 24 × 60 mm glass cover slides (Menzel-Gläser, Braunschweig, Germany) and Loctite EA M-31 CL (Henkel Adhesives, Nieuwegein, The Netherlands).

After construction, the HFM was sterilized in 70% (v/v) EtOH for 30 min, washed in PBS, incubated in L-3,4-di-hydroxy-phenylalanine (L-DOPA, 2 mg/mL in 10 mM Tris-HCl buffer, pH 8.5) at 37 °C 5% CO_2_ for 5 h, and washed again in PBS. To finalize the ECM-coating, HFM was exposed to human collagen IV (25 µg/mL in PBS) at 37 °C and 5% CO_2_ for 2 h. After removing the human collagen IV solution, HFM was kept in PBS at 37 °C 5% CO_2_ awaiting cell seeding. Caco-2 cells were seeded on HFM at a density of 1.0 × 10^6^ cells/fiber and cultured for 21 days to form a bioengineered intestinal tubule. This tubule was exposed to a physiological relevant flow (0.006 dyne/cm^2^) on a 2-dimensional plate rocker at 10° at 1 rotation per minute (VWR, Breda, The Netherlands) during the final 7 days.

### Plant cultivation and exposure to plant extracts

To validate the bioengineered intestinal tubule, butterhead, red leaf lettuce (type Lollo Rossa), red crisphead, and stalk lettuce were purchased freshly at a local supermarket (Utrecht, The Netherlands). To evaluate the effect of extracts from diverse lettuce germplasm pools, plants of *L. sativa* cv. Salinas (CGN25281) and cv. Olof (CGN05786) (both GP1), *L. serriola* US96UC23 (GP1; CGN25282), *L. saligna* CGN05271 (GP2), *L. virosa* CGN04683 (GP3) were grown for 2.5–3 weeks under the 16 h/8 h light regime at ~200 µmol/sec/m^2^ (LED white light), with temperature 21 °C (light phase) or 19 °C (dark phase) and relative humidity 70%. Seed materials are available from the Center for Genetic resources Netherlands (CGN, Wageningen, the Netherlands). Leaves of most lines or the stem of stalk lettuce were snap-frozen in liquid nitrogen, ground to a fine powder, and stored at −80 °C until exposure. Bioengineered intestinal tubules were exposed to the unfiltered extracts at a 0.5 g/mL concentration in a culture medium for 24 h. After the exposure, the supernatant was collected and stored at −20 °C until further analysis. After collecting the supernatant, bioengineered intestinal tubules were evaluated for epithelial barrier integrity, cell viability, cell attachment, and alkaline phosphatase activity.

### Inulin-FITC leakage assay

Bioengineered intestinal tubules were washed three times with 1xPBS at room temperature to remove of remaining lettuce extracts and particles. Then, bioengineered intestinal tubules were connected to a Reglo Independent Channel Control pump (Ismatec, Wertheim, Germany) and perfused at 0.1 mL/min for 10 min with 0.1 mg/mL inulin-FITC solution (#F3272-1G). The amount of inulin-FITC transferred into the chamber was measured using GloMax® Discover (Promega, Leiden, The Netherlands) set at excitation wavelength 475 nm and emission wavelengths 500–550 nm. The emission intensities were normalized relative to unseeded bioengineered intestinal tubules set as 100% permeable and 0% epithelial barrier integrity. Next, bioengineered intestinal tubules were washed with 4% FCS in HBSS (v/v), removed from the chamber, and cut into two parts. The first part was used for immunofluorescent staining, and the second fragment was subjected to PrestoBlue™ staining and alkaline phosphatase activity assay.

### Immunofluorescent staining

The bioengineered intestinal tubules were immunofluorescently stained for the goblet cell marker mucin-2 (MUC2) to assess cell differentiation, zonula occludens-1 (ZO-1) to examine the tight junction expression between cells, and 4′,6-diamidino-2-fenylindool (DAPI) to count the number of cells. Bioengineered intestinal tubules were fixed for 5 min (60% EtOH, 30% chloroform and 10% acetic acid (v/v)), permeabilized for 10 min (0.3% (v/v) Triton X-100 in HBSS) and blocked for 30 min in blocking buffer (2% (w/v) bovine serum albumin fraction V with 0.1% (v/v) Tween-20 in HBSS). Next, cells were exposed to primary antibodies against MUC2 (1:200, #ab118964 Abcam, Cambridge, United Kingdom) and ZO-1 (1:1000, #40-2200, Thermo Fisher Scientific, Bleiswijk, The Netherlands) in a blocking buffer for 2 h. After washing in 1× PBS, the cells were incubated with a secondary antibody, donkey-anti-rabbit Alexa Fluor 488 (1:200, Thermo Fisher Scientific), goat-anti-mouse Alexa Fluor 488 (1:200, Thermo Fisher Scientific) and/or goat anti-rabbit Alexa Fluor 594 (1:200, #ab150084, Abcam), diluted in blocking buffer for 1 h. Finally, the cells were washed with 1× PBS and mounted using Prolong gold-containing DAPI (Cell signaling technology, Leiden, The Netherlands). For each bioengineered intestinal tubule, three full fields of view z-stacks were acquired at random spots using a Leica TCS SP8 X system (Leica Biosystems, Amsterdam, The Netherlands).

Image analysis was done in Fiji ImageJ version 2.0.3 as previously described^5^. In short, a z-stack was transformed into a maximum intensity projection. Channels were separated, and a custom region of interest (ROI), consisting of 28 horizontal lines, was applied to the channel containing the ZO-1 staining. The number of intersections between a positive ZO-1 staining and a horizontal line was determined using the Peakfinder tool with a tolerance set at twice the determined noise level. The channel containing the DAPI staining was used to count the number of cells using the analyze particle function. After analysis, the number of cells was corrected for surface area, and the ZO-1 was corrected for the number of cells and surface area.

### Cell viability

The mitochondrial activity assessed by the PrestoBlue™ solution (#A13261, Thermo Fisher Scientific) was used to evaluate cell viability. A total of 100 µL of the reagent diluted 1:10 in the cultivation medium was added to one part of the bioengineered intestinal tubule and incubated at 37 °C 5% CO_2_ for 1 h, protected from light. Fluorescence resulting from the reduction of PrestoBlue™ was measured on GloMax® Discover (Promega) set at 520 nm excitation and 580–640 nm emission wavelengths. Values were corrected for bioengineered intestinal tubule length, and measured values were calculated relative to the medium-only control.

### Alkaline phosphatase activity assay

The Amplite™ Colorimetric Alkaline Phosphatase Activity Assay (AAT Bioquest, Sunnyvale, United States) was performed according to the manufacturer’s protocol. In short, bioengineered intestinal tubules were washed in PBS and incubated in p-nitrophenyl phosphate (pNPP) working solution (50:50 (v/v) in PBS) for 30 min protected from light at 37 °C and 5% CO_2_. After 30 min, light absorbance was measured at 405 nm using GloMax® Discover (Promega). Activity values were divided by mm bioengineered intestinal tubule to correct for tubule length.

### IL-6 and IL-8 secretion assay

Stored supernatants were thawed and centrifuged for 5 min at 18213 rcf to reduce interference from plant residues. IL-6 and IL-8 were quantified by ELISA (Biolegend, London, UK) according to the manufacturer’s protocol. Plates were coated and incubated overnight, followed by blocking for 1 h. The coated plates were exposed to the supernatants and incubated for additional 2 h. This was followed by incubation with the detection antibody for 1 h and Avidin-HRP for 30 min. Finally, wells were incubated with the substrate solution for 15 min. After the addition of the stop solution, absorbance was measured using GloMax® Discover at 450 nm. Cross-reactivity was also determined in plant extracts not added to bioengineered intestinal tubules.

### Nitric oxide (NO) content

Supernatant samples were centrifuged for 5 min at 18213 rcf to remove plant debris, and NO content was determined by Griess reaction (Promega) according to the manufacturer’s protocol. Sulfanilamide solution was added to the wells and incubated for 10 min, followed by 10 min incubation with N-1-naphthylethylenediamine dihydrochloride (NED) solution to reach a total ratio of 50:50 (v/v). The absorbance at 490 nm was measured on GloMax® Discover (Promega). In addition, NO content was determined in plant extracts.

### Statistical and comprehensive cluster analysis

GraphPad version 8 was used for data analysis. First, data were tested for outliers using the ROUT method with Q = 1%. Data sets were tested for significance using t-test and one-way ANOVA with a *P*-value of < 0.05 considered significant.

The data were analyzed further with the comprehensive cluster analysis. First, the mean value per plant line per measured parameter was calculated for each experimental run. These values were subsequently scaled to the 0–1 range. The data were then clustered with the K-means algorithm in Python scikit-learn package. To determine an appropriate number of clusters, the algorithm was run with *k* 1 to 11, and the knee point on the sum of squared distances vs. the cluster number *k* was determined with the KneeLocator function (Python, kneed package). Additionally, to justify the choice of *k*, the silhouette coefficient was computed for models with 1 to 11 clusters as the difference between the mean intra-cluster and inter-cluster distances divided by the highest of these two means.

### Reporting summary

Further information on research design is available in the Nature Research Reporting Summary linked to this article.

## Supplementary information

Supplementary Material

Reporting Summary

Supplementary Figures

## Data Availability

The data that support the findings of this study are available from the corresponding author. Used plant accession can be requested from the Center for Genetic resources in the Netherlands (CGN): CGN25281, CGN05786, US96UC23 [https://cgngenis.wur.nl/accessiondetails/CGN25282], CGN05271, CGN04683.

## Code availability

Custom code is available at https://github.com/NaomiMoonen/ClusteringLettuceSpecies.
