# Supplementary figures and images for: Bioengineered intestinal tubules as a tool to test intestinal biological efficacy of lettuce species
Source: NPJ Sci Food. 2022 Dec 13;6:58. doi: 10.1038/s41538-022-00175-x (PMC9747904; doi:10.1038/s41538-022-00175-x)

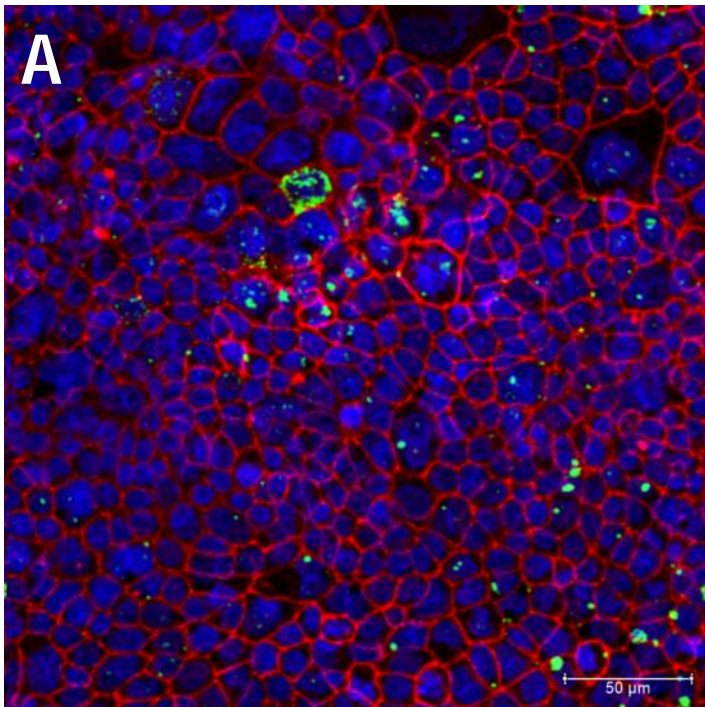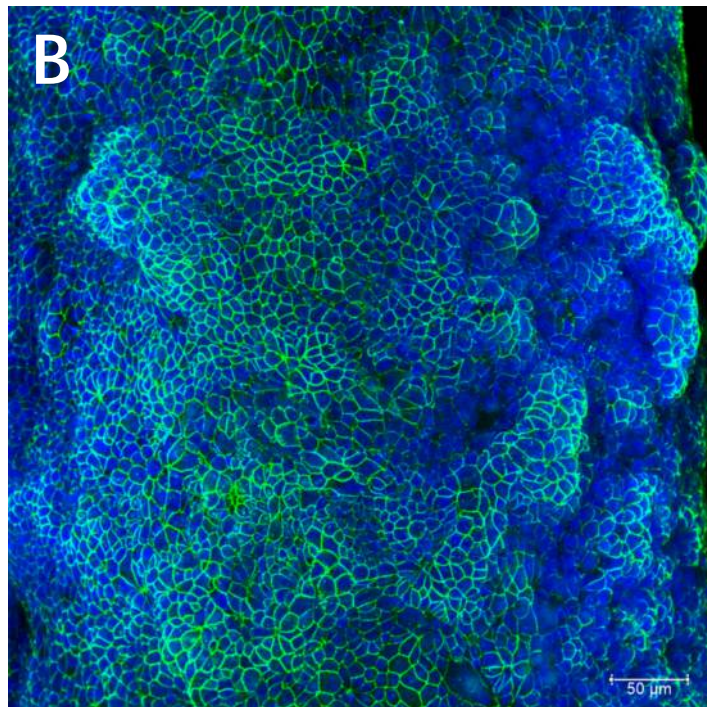

Supplement: Supplementary file 1 — Supplementary Material [file 41538_2022_175_MOESM1_ESM.pdf]
