# Supplementary material for: Bioengineered intestinal tubules as a tool to test intestinal biological efficacy of lettuce species
Source: NPJ Sci Food. 2022 Dec 13;6:58. doi: 10.1038/s41538-022-00175-x (PMC9747904; doi:10.1038/s41538-022-00175-x)
Supplement: Supplementary file 3 — Supplementary Figures [file 41538_2022_175_MOESM3_ESM.docx]

**Supplementary Figures**

**Supplementary figure 1.** **Morphological structures in engineered intestinal tubules.** (a) Shows a representative immunofluorescent image of a bioengineered intestinal tubule stained for zonula occludens-1 (ZO-1, red), Mucin-2 (MUC2, green) and nuclei (blue). (b) Shows a representative 3-dimensional composition of an immunofluorescent image of the bioengineered intestinal tubule stained for ZO-1 (green) and nuclei (blue). Scale bar is 50 uM.
